# Complement C5a Induces Pro-inflammatory Microvesicle Shedding in Severely Injured Patients

**DOI:** 10.3389/fimmu.2020.01789

**Published:** 2020-09-02

**Authors:** Ebru Karasu, Julia Demmelmaier, Stephanie Kellermann, Karlheinz Holzmann, Jörg Köhl, Christoph Q. Schmidt, Miriam Kalbitz, Florian Gebhard, Markus S. Huber-Lang, Rebecca Halbgebauer

**Affiliations:** ^1^Institute of Clinical and Experimental Trauma Immunology, University Hospital Ulm, Ulm, Germany; ^2^Center for Biomedical Research, Genomics-Core Facility, Ulm University, Ulm, Germany; ^3^Institute for Systemic Inflammation Research (ISEF), University of Lübeck, Lübeck, Germany; ^4^Division of Immunobiology, Cincinnati Children's Hospital, Cincinnati, OH, United States; ^5^Institute of Pharmacology of Natural Products and Clinical Pharmacology, Ulm University, Ulm, Germany; ^6^Department of Traumatology, Hand-, Plastic- and Reconstructive Surgery, Center of Surgery, University of Ulm Medical School, Ulm, Germany

**Keywords:** multiple trauma, microvesicle shedding, anaphylatoxin C5a, C5aR1, neutrophils, polymorphonuclear neutrophils (PMNs), inflammation

## Abstract

Initially underestimated as platelet dust, extracellular vesicles are continuously gaining interest in the field of inflammation. Various studies addressing inflammatory diseases have shown that microvesicles (MVs) originating from different cell types are systemic transport vehicles carrying distinct cargoes to modulate immune responses. In this study, we focused on the clinical setting of multiple trauma, which is characterized by activation and dysfunction of both, the fluid-phase and the cellular component of innate immunity. Given the sensitivity of neutrophils for the complement anaphylatoxin C5a, we hypothesized that increased C5a production induces alterations in MV shedding of neutrophils resulting in neutrophil dysfunction that fuels posttraumatic inflammation. In a mono-centered prospective clinical study with polytraumatized patients, we found significantly increased granulocyte-derived MVs containing the C5a receptor (C5aR1, CD88) on their surface. This finding was accompanied by a concomitant loss of C5aR1 on granulocytes indicative of an impaired cellular chemotactic and pro-inflammatory neutrophil functions. Furthermore, *in vitro* exposure of human neutrophils (from healthy volunteers) to C5a significantly increased MV shedding and C5aR1 loss on neutrophils, which could be blocked using the C5aR1 antagonist PMX53. Mechanistic analyses revealed that the interaction between C5aR1 signaling and the small GTPase Arf6 acts as a molecular switch for MV shedding. When neutrophil derived, C5a-induced MV were exposed to a complex *ex vivo* whole blood model significant pro-inflammatory properties (NADPH activity, ROS and MPO generation) of the MVs became evident. C5a-induced MVs activated resting neutrophils and significantly induced IL-6 secretion. These data suggest a novel role of the C5a-C5aR1 axis: C5a-induced MV shedding from neutrophils results in decreased C5aR1 surface expression on the one hand, on the other hand it leads to profound inflammatory signals which likely are both key drivers of the neutrophil dysfunction which is regularly observed in patients suffering from multiple traumatic injuries.

## Introduction

In patients with severe injury, sepsis, and septic shock (1, 2) early and excessive complement activation may be responsible for subsequent innate immune dysfunction (1, 3, 4). Neutrophils as first-line cellular defense of innate immunity showed major changes in their function driven by an imbalanced complement activity. Equipped with a broad receptor repertoire for complement activation products including the anaphylatoxin receptors as well as membrane-bound complement regulators, neutrophils quickly sense micro-environmental changes. Our previous study showed altered expression profiles of complement receptors and membrane-bound complement regulators on immune cells from polytraumatized patients (5). So far, it is well-established that the anaphylatoxin C5a exerts its effects through the two complement anaphylatoxin C5a receptors, C5aR1 (CD88) and C5aR2. While C5aR1 is a classical G-protein coupled receptor, C5aR2 is deficient in G-protein coupling. Especially, dysfunction of C5aR1 has been implicated in various pathological conditions such as sepsis, autoimmunity and inflammation (6–8). Neutrophils, as first cellular line of defense, constitutively express C5aR1 (9), which represents the major receptor for the anaphylatoxin. C5aR1 acts as switch to induce multiple functions of neutrophils including chemotaxis, adhesion, intracellular pH changes and electrophysiological changes (10–12). C5aR1 dysfunction negatively influences cellular effector functions and is associated with poor clinical outcome in sepsis patients (3, 13–15).

There is increasing evidence that MVs are involved in the loss of cellular C5aR1. In a previous study, we showed a significant reduction of the C5aR1 on neutrophils from septic shock patients and simultaneously increased plasma levels of a circulating form of C5aR1, suggesting that cells lose their receptors through MV shedding (3). However, a direct linkage among C5aR1 loss and MV shedding has not been demonstrated so far. MV shaping involves a dynamic interplay between phospholipid redistribution and cytoskeletal proteins. MV formation is induced by translocation of phosphatidylserine to the outer membrane through the activity of aminophospholipid translocases. It is well-established that the small and ubiquitously expressed GTP-binding proteins ADP-ribosylation factor (Arf) proteins possess important roles in membrane trafficking including vesicle formation (16, 17). In principle, MVs can transfer information from the MV-generating donor cell to a wide range of remote target cells (18). Besides communication and transport vehicles, MVs are highly relevant in the context of inflammation. These packages actively contribute in inflammatory processes by interacting with immune cells, epithelial and endothelial cells, and bridging innate and adaptive immunity (19, 20). Several studies showed that MV numbers and their origin are altered in trauma and sepsis (21–23). Patients with major burn injury showed elevated numbers of granulocyte- and monocyte- derived MVs, and this increase was associated with injury severity (24).

In our present study, we focused on the anaphylatoxin C5a in MV shedding, because (i) increased concentrations are present early after multiple trauma and correlates with clinical severity, and (ii) the C5a-C5aR1 axis has already been shown to lead to a paralyzed innate immune response but underlying mechanisms remain mostly unexplained. We hypothesized that a link between C5aR1 signaling and Arf6-mediated MV shedding in neutrophils is responsible for altered C5aR1 levels on neutrophils under systemic inflammatory conditions.

## Materials and Methods

### Clinical Study on Polytraumatized Patients

A prospective clinical study was conducted in patients after severe trauma (Injury Severity Score ≥ 32, *n* = 11) and healthy volunteers of similar sex and age distribution (*n* = 8) (Table 1). The study was approved by the Independent Local Ethics Committee of the University of Ulm (number 94/14). Written informed consent was collected for each subject. Blood was obtained upon admission to the emergency room (ER) or 0 h, and 4, 12, 24, 48, 120, and 240 h after trauma. Citrate and EDTA blood from PT patients were centrifuged at 800 × g for 15 min at 4°C, followed by a second centrifugation at 16,000 × g for 2 min at 4°C. The supernatant was transferred into a new tube and was stored at −80°C.

**Table 1:** Study participants.

	**PT (*n* = 11)**	**Healthy (*n* = 8)**	
**Demographic data**	**Mean** **±** **SEM;**	**Mean** **±** **SEM;**	***p*****-value**
	**median**	**median**	
	**(min.–max.)**	**(min.–max.)**	
Age (years)	44.5 ± 3.9; 49 (21–63)	42.4 ± 5.4; 42 (25–64)	n.s.
Sex, m/f (n)	9/2	5/3	n.s.
**Injury severity and clinical course**			
ISS	35.9 ± 1.5; 34 (27–43)	n/a	
GCS	4.9 ± 1.2; 3 (3–15)	n/a	
ICU stay (d)	10.4 ± 3.0; 10 (1–32)	n/a	
Death, *n* (%)	4 (36%)	n/a	
Nosocomial infection, *n* (%)	6 (54%)	n/a	
**Mechanism of injury**			
Car crash *n* (%)	5 (45%)	n/a	
Bicycle—car collision *n* (%)	3 (27%)	n/a	
Fall *n* (%)	3 (27%)	n/a	
**Clinical parameters**			
Systolic RR (mmHg) min/max	65.9/103.3 ± 6.1/6.2;65/110 (40/60–110/120)	n/a	
BE	−1.7 ± 0.8; −2.7 (-4.8–3)	n/a	
Initial Lactate (mmol/l)	2.2 ± 0.3; 1.7 (0.8–3.7)	n/a	
INR	1.4 ± 0.1; 1.3 (0.9–2.8)	n/a	
Initial Quick (%)	65.3 ± 10.8; 58 (24–124)	n/a	
Initial PTT (sec)	40.1 ± 4.9; 37 (21–77)	n/a	
RBC transfusion 0 ≥ 24 h (unit)	3.8 ± 1.43; 2 (0–15)	n/a	
TASH score	8.4 ± 1.0; 8 (2–13)	n/a	
Initial leukocytes (cells/nl)	14.3 ± 1.9; 14 (4.9–26.1)	n/a	

*Central clinical data of study participants. Ages were compared using Student's t-test, and sexes were compared using Fisher's Exact test*.

*BE, base excess, GCS, Glasgow Coma Scale, ICU, intensive care unit, INR, international normalized ratio; ISS, injury severity score, PT, polytrauma; PTT, partial thromboplastin time; RBC, red blood cell; RR, Riva-Rocci, SEM, standard error of the mean; TASH score, trauma associated severe hemorrhage score*.

### Microvesicle Enrichment in Plasma Samples

For flow cytometric analyses of CD66b and C5aR1 on the surface, MVs were enriched in patients' plasma samples. Plasma was thawed and centrifuged at 20,000 × g for 45 min. Subsequently, the pelleted MV fraction was resuspended in Annexin V-binding buffer (BD Pharmingen) and stored at −80°C until further analysis.

### MV Flow Cytometric Analysis

For identification of MVs, flow cytometry (FACSCanto II, BD) was used to define a MV-specific gate by size calibration beads and presence of Annexin V (AV). AV binding buffer was filtered twice (0.2 μm pore membrane filter). Five microliter of enriched or non- enriched plasma samples were used per measurement. MVs were stained with mouse anti-human C5aR1-FITC antibody (MCA2059F, Bio-Rad) and mouse anti-human CD66b-APC-Cy7 (305126, Biolegend) or the respective isotype controls in AV binding buffer. Gates to define size were set using 0.3, 0.5, and 1 μm latex beads (latex beads polystyrene 0.3 μm Kat: LB3-1ML; latex beads, carboxylate-modified polystyrene, fluorescent red, 0.5 μm, Kat: L3280-1ML; latex beads, amine-modified polystyrene, fluorescent red 1.0 μm, Kat: L2778-1ML; all Sigma Aldrich). The lowest detection limit for digital flow cytometry based on size calibration beads is 0.2 μm; since MVs are defined as AV-positive vesicles ranging from 0.2 to 0.9 μm, the MV gate was set at this limit. Gate borders of Q1 to Q4 were defined according to isotype controls. A known quantity of counting beads of a 4.2 μm diameter (C36950, Thermo Fisher Scientific) was used and the absolute number of MV per plasma volume was calculated based on counting beads using the following formula: MV/μl = (MV count/bead count) × (total number of beads/test volume).

### PMN Isolation and Stimulation

After written informed consent was collected human blood was drawn by peripheral venous puncture into sodium citrate monovettes (approval by the Local Independent Ethics Committee of the University of Ulm (number 459/18 and 462/18). Subjects were healthy males and females between 18 and 35 years without signs of infection or any current medical problems or medication. PMNs were isolated from whole blood by Ficoll-Hypaque gradient centrifugation (GE Healthcare) and dextran sedimentation (Dextran from Leuconostoc spp.; Sigma), followed by hypotonic lysis of residual red blood cells. PMNs were counted and adjusted to 5 × 10^6^ cells/mL in HBSS++ (Gibco) or in RPMI buffer (Gibco). PMNs were incubated with 100 ng/ml C5a (Complement Technology, USA) or human C5a^desArg^ (Sigma, USA), human C3a (Complement Technology), 500 pg/ml human recombinant IL-6 (Biomol), 200 pg/ml human recombinant IL-8 (Biomol), 150 pg/ml human recombinant IL-1β (PeproTech), or 5 μg/ml LPS (Sigma, USA). Untreated cells served as controls. To further investigate the C5a-mediated effects, we used 10 μM of the selective small peptide C5aR1 antagonist PMX53 (kind gift of John Lambris, Department of Pathology and Laboratory Medicine, University of Pennsylvania School of Medicine, United States) and the Arf6-selective inhibitor NAV2729 (5986, Tocris). All experiments were performed on a rotating wheel at 37°C and 80 rpm; after incubation, cells were centrifuged at 340 × g for 5 min. Supernatants were stored at −80°C for MV analysis. The pelleted cells were washed with PBS twice, stained with anti-human C5aR1 antibody (1:100 dilution) for 20 min at 4°C, washed and fixed with CellFix (BD) and analyzed by flow cytometry after gating based on light scatter characteristics.

Moreover, PMNs from healthy volunteers were incubated in HBSS++ containing 20% of human serum from either polytraumatized patients or from healthy donors. For this purpose, sera from three patients or three healthy donors were pooled, respectively. Since PMN supernatants were analyzed for MV shedding, we removed MVs in sera by centrifugation at 20,000 × g at 4°C for 45 min. PMNs were incubated for 1 h at 37°C.

For further *in vitro* stimulation experiments, the supernatant after PMN stimulation with 100 ng/ml C5a ± 10 μM C5aR1 antagonist was centrifuged at 3,000 × g for 10 min; MVs were pelleted by centrifugation at 20,000 × g for 45 min. The supernatant was discarded and the MV–containing pellet was washed twice with PBS and centrifuged again at 20,000 × g for 45 min. MVs were resuspended in PBS and stored at −80°C. PMNs were stimulated with the generated MV from autologous donors for 1 h at 37°C. Subsequently functional PMN assays including NADPH oxidase activity, ROS generation, and MPO release were investigated.

### Western Blotting

PMNs were resuspended in cold RIPA buffer containing a protease- and phosphatase inhibitor cocktail (Thermo Fisher). Cells were gently resuspended, sonicated and stored on ice for 20 min. After a second sonication step, 4x Laemmli buffer including beta-mercaptoethanol (Sigma) was added. Samples were denatured for 5 min at 95°C. For electrophoresis, precast 4–20% gradient gels (Bio-Rad) were used. After electrophoresis, proteins were transferred on a PDVF membrane (GE Healthcare) and membranes were blocked in BSA/TBST for 1 h. Subsequently, membranes were incubated with rabbit anti-human phospho-p47^phox^ (Ser345) antibody (Invitrogen, 1:500 dilution in BSA/TBST) overnight at 4°C. After washing, membranes were incubated with anti-rabbit-IgG-HRP secondary antibody (Cell Signaling, 1:1500 dilution in BSA/TBST). Western clarity ECL solution (Bio-Rad) was used for development. A Chemidoc XRS+ (Bio-Rad) was used for detection of bands. Protein expression was normalized to total protein using ImageLab (Bio-Rad). For C5a immunoblots, washed MV from PMN supernatants were equally loaded on 4–20% gradient gels. After protein transfer, PVDF membranes were blocked in 5% milk/TBST and incubated with the primary antibody rabbit anti-human C5a (Calbiochem) overnight at 4°C. After washing, membranes were incubated in anti-rabbit-IgG-HRP secondary antibody (Cell Signaling, 1:1500 dilution in 5% milk/TBST).

### ROS Detection

Isolated PMNs were adjusted to 5 × 10^6^ cells/mL in HBSS++ and were incubated with Dihydrorhodamine (DHR) 123 (Santa Cruz, 1:1000 dilution) at 37°C for 30 min protected from light. After incubation, cells were washed and resuspended in HBSS++ containing 0.1% BSA (Sigma). PMNs were seeded on 96-well plates and incubated for 10 min at 37°C. After incubation, baseline fluorescence was determined with a fluorescence reader (Fluoroskan Ascent, Thermo Fisher). PMNs were stimulated with 5 μl MV preparations and incubated at 37°C protected from light. Measurements were performed directly, 10, 30, 60, and 120 min after incubation.

### Myeloperoxidase Assay

For assessment of myeloperoxidase (MPO) activity, neutrophil supernatant or standard human MPO (Merck) was incubated with tetramethylbenzidine (100 μg/ml) and H_2_O_2_ (0.0016%) at 37°C. After 5 min, the reaction was terminated by the addition of 2 M H_2_SO_4_, and the absorbance at 450 nm was determined using a spectrophotometric reader (Sunrise, Tecan).

### Whole Blood Model and Experiments

For *ex vivo* experiments, we used a recently published human whole blood model employing lepirudin at a final concentration of 50 μg/ml; in contrast to heparin, lepirudin does not inhibit complement activation (25). For whole blood model experiments, PMN-derived MVs were added to 1 ml of whole lepirudin blood from autologous donors and incubated at 37°C and 80 rpm. After incubation for 1 and 4 h, this reaction was supplemented with 10 mM EDTA and plasma was obtained by centrifugation at 800 × g for 15 min followed by a second centrifugation step at 16,000 × g for 2 min to remove cellular debris. Plasma was stored at −80°C until ELISA analysis. Human IL-6 ELISA (R&D Systems) was performed with plasma samples according to the manufactures' instructions.

### RNA Isolation and Microarray Analysis

Since eosinophils are more transcriptionally active cells than neutrophils and thus can affect transcriptome analysis (26), we performed depletion of eosinophils with CD9-selective beads (Miltenyi) to gain higher purity of neutrophils. Purified neutrophils were untreated or C5a-stimulated for 1 h at 37°C on a rotating wheel. After stimulation, RNA extraction was performed by Guanidinium Thiocyanate–Phenol–Chloroform (TRIZOL) and resuspended in nuclease-free water. Microarray analyses were performed using 200 ng total RNA as starting material and 5.5 μg ssDNA per hybridization (GeneChip Fluidics Station 450; Affymetrix, Santa Clara, CA). Total RNAs were amplified and labeled following the Whole Transcript (WT) Sense Target Labeling Assay (http://www.affymetrix.com). Labeled ssDNA was hybridized to Human Gene 1.0 ST GeneChip arrays (Affymetrix, Santa Clara, CA). The chips were scanned with a Affymetrix GeneChip Scanner 3000 and subsequent images analyzed using Affymetrix® Expression ConsoleTM Software (Affymetrix).

Transcriptome analysis: Transcriptome analysis was performed using BRB-ArrayTools developed by Dr. Richard Simon and BRB- ArrayTools Development Team (http://linus.nci.nih.gov/BRB-ArrayTools.html). Raw feature data were normalized and log 2 intensity expression summary values for each probe set were calculated using robust multiarray average (27). Filtering: Genes showing minimal variation across the set of arrays were excluded from the analysis. Genes whose expression differed by at least 1.5-fold from the median in at least 20% of the arrays were retained.

Class comparison: Genes were identified as differentially expressed among the two classes using a 2 sample *t*-test. Genes were considered statistically significant if their *p* < 0.05 (FDR < 0.1) and displayed a fold change between the two groups of at least 1.5-fold. Benjamini and Hochberg correction was used to calculate the false discovery rate (28). Complete microarray data are available at Gene Expression Omnibus (GEO accession number: GSE150902).

### Gene Ontology Analysis

Gene Ontology Analysis of differentially expressed genes: To identify the most affected biological processes, as defined by Gene Ontology annotation, we used the GoMINER analysis tool (29). This package allows the automatic analysis of multiple microarrays and then integrates the results, across all of them, to find the GO categories that were significantly over- or under-represented.

### Generation of Heat Maps

A heat map was designed with selected, and MV-relevant genes using Genesis (Alexander Sturn and Rene Snajder, TU Graz, version 1.8.1). Gene expression was normalized, sorted by trend of expression value and displayed with a set upper maximum value of +0.9 and a lower maximum value of −0.9.

### Statistical Analysis

All results are shown as mean ± SEM. Groups were compared using one-way ANOVA if not indicated otherwise. Student-Newman-Keuls *post hoc* testing was performed in SigmaPlot (Version 14, Systat, Germany). Results were considered statistically significant where *p* < 0.05.

## Results

### MV Detection by Flow Cytometry

**Figure 1:**
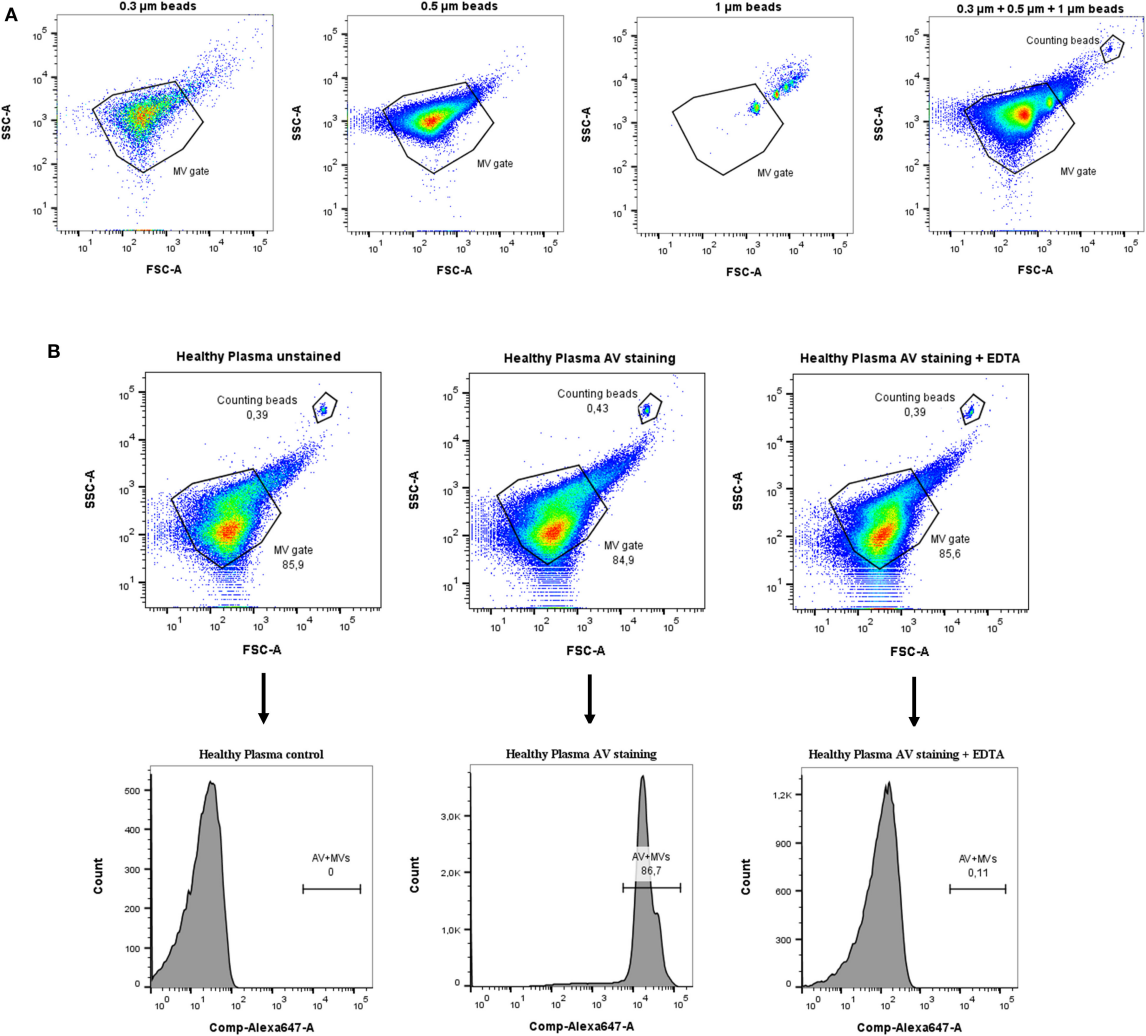
Gating strategy for detection of MVs in plasma samples. **(A)** Size calibration beads ranging from 0.3 to 1 μm were used to define a MV gate. **(B)** AV staining was further included to specifically detect MVs due to their PS surface expression. EDTA control was included to block specific binding of AV to PS. Counting beads were used to quantify the amount of shed MVs in samples as described in the text.

The size of MVs is described to range between 100 nm to 1 μm. To exclude contaminant exosomes (50–100 nm) and apoptotic bodies (>1 μm) in our samples, we defined the MV-specific gate by using size calibration beads ranging from 0.3 to 1 μm (Figure 1A). Moreover, to quantify MV counts, plasma samples included a known number of counting beads *per set* volume, which were used to stop sample analysis after 500 events allowing a quantification of MV counts per plasma volume. Since phosphatidylserine (PS) is an established marker for MVs, samples were stained with AV, which binds calcium-dependently to PS. Calcium chelation by EDTA served as control of AV specificity (Figure 1B).

### Post-traumatic C5aR1 Expression Kinetics on Neutrophil and MV Surfaces

**Figure 2:**
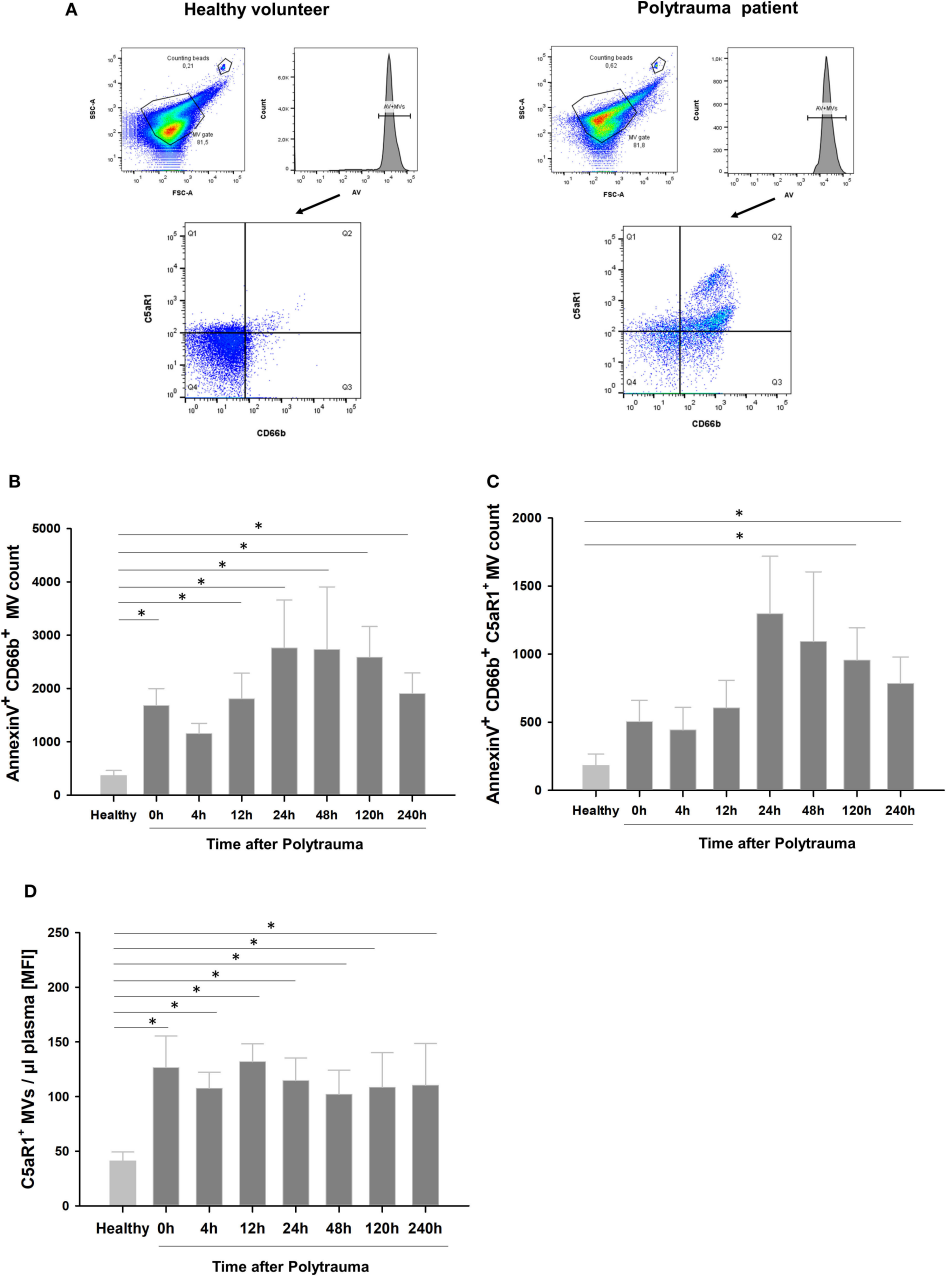
Expression profile of shed MVs in multiple trauma. **(A)** Representative dot plot of a healthy control (left) and a multiple trauma patient (right). Annexin V- positive MV fraction was analyzed for CD66b and C5aR1 expression. FITC was used for C5aR1 staining, and APC-Cy7 for CD66b. Q2 represents AnnexinV^+^, C5aR1^+^, CD66b^+^ MVs positive MV fraction. **(B)** Quantitative analysis for granulocyte-derived MVs and **(C,D)** granulocyte-derived MVs expressing C5aR1 in 5 μl of MV enriched plasma, respectively. *n* = 8–10, *p < 0.05 compared to healthy.

Figure 2A shows representative samples of a healthy donor and a multiple injured patient, respectively. AV^+^ MV fraction was determined from the MV gate and used for further analysis. For AV^+^ MVs, Q2 contains CD66b^+^C5aR1^+^ MVs, while the sum of Q2+Q3 represents the total amount of PMN-derived MVs. Quantitative analysis revealed that plasma samples from patients after polytrauma showed a significant time-dependent increase of granulocyte-derived MVs compared to healthy controls (Figure 2B). Furthermore, we detected significantly more C5aR1^+^ neutrophil-derived MVs after trauma and C5aR1 levels on these MVs were significantly increased at the time points 24 and 120 h, respectively (Figure 2C), while MFI values were significantly increased at all time points after PT (Figure 2D). In concordance, PMNs from these patients showed a significant and time-dependent reduction of the C5aR1 (Supplemental Figure 1A) in line with our previous study (5).

### C5a Induces Shedding of PMN-Derived MVs *in vitro*

**Figure 3:**
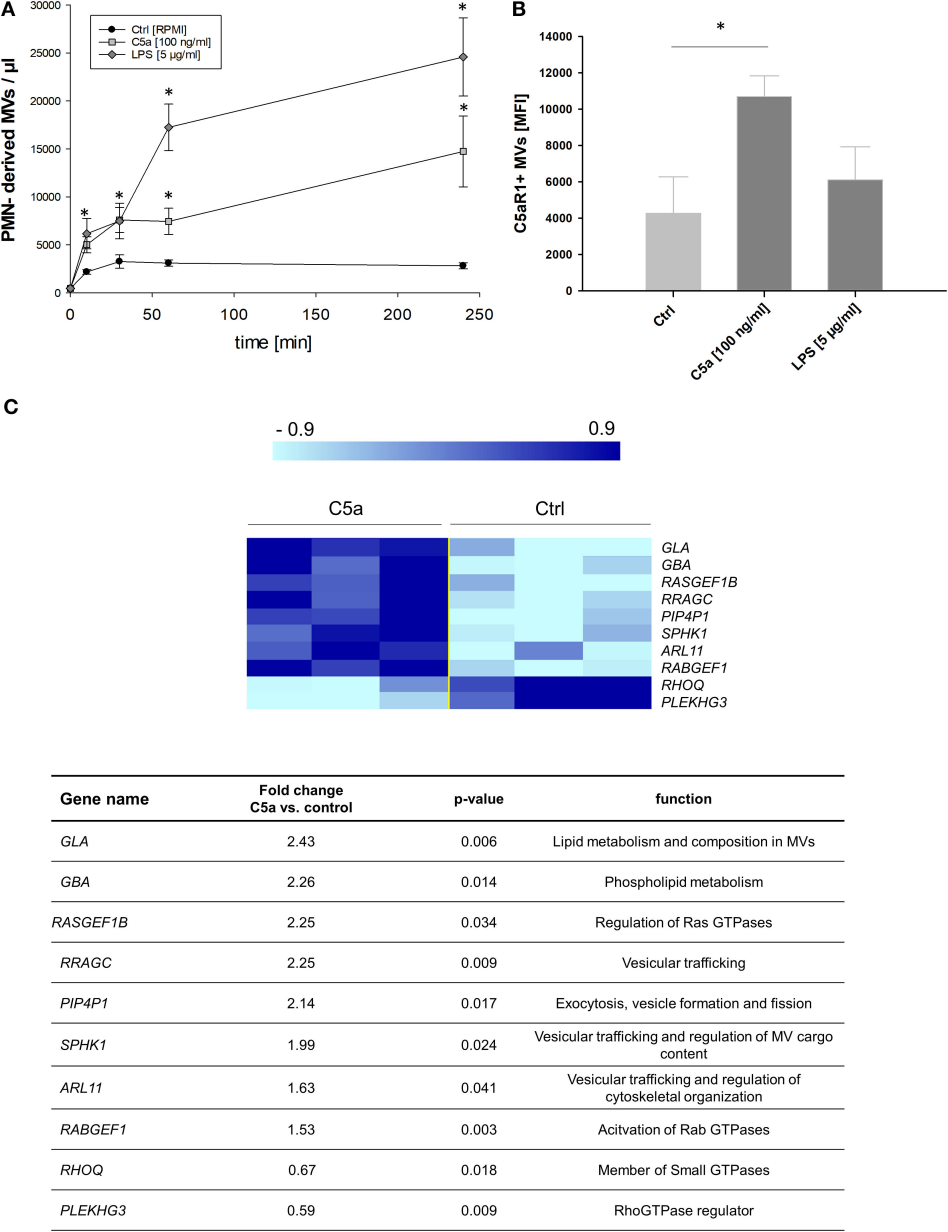
C5a alters MV shedding in PMNs. **(A)** PMNs were stimulated for 0, 10, 30, 60, and 240 min with C5a (100 ng/ml) or LPS (5 μg/ml) and supernatants were analyzed by FACS. Untreated cells served as control. *n* = 10, One-Way ANOVA followed by Student-Neuman-Keuls *post-hoc* testing was performed for each time point, *p < 0.05. **(B)** MVs from supernatants were further analyzed for expression of C5aR1 by FACS. *n* = 3 per group, *p < 0.05. **(C)** Heat map and gene list of selected and differentially regulated genes upon C5a treatment in in neutrophils after 1 h (*n* = 3 per group).

After severe trauma, there is an excessive complement activation and C5a generation (1), and we investigated whether C5a stimulation of neutrophils results in shedding of MVs. While resting cells showed the lowest amount of MVs, C5a significantly increased MV shedding in a time-dependent manner (Figure 3A). LPS, which has been described as a potent inducer of MV shedding in neutrophils (30), resulted in an even stronger induction of PMN-derived MVs compared to C5a (Figure 3A). Characterization of the MVs showed that C5a induced shedding of C5aR1^+^ MVs, which was not observed for LPS (Figure 3B). To further compare C5a with other pro-inflammatory mediators, we incubated neutrophils with trauma-relevant concentrations of IL- 6, IL-1β, IL-8, and C3a. The shedding of PMN-derived MVs and C5aR1 on MVs was specific to C5a and was not observed for any of the other cytokines and chemokines (Supplemental Figure 2A).

To identify the underlying mechanisms, we performed a microarray analysis of gene expression in C5a-stimulated neutrophils. Figure 3C illustrates a heat map of selected genes. We found significant alterations in genes associated with generation of extracellular vesicles including small GTPases and genes encoding for regulatory proteins of GTPases and Arf proteins; fold change expression patterns in response to C5a are listed in the lower panel of Figure 3C. While the microarray experiment does not show a significant change in expression of classical MV-forming proteins including Arf1, Arf6, and Rab22 GTPases, the expression of other important members of small GTPases was significantly increased including Ras homolog family member Q (RHOQ), ADP ribosylation factor like GTPase 11 (Arl11), and Ras related GTP binding C (RRAGC). We also found significant changes in the expression of genes described to regulate GTPase activity. Among them, we identified increased expression in RAB guanine nucleotide exchange factor 1 (RabGEF1), pleckstrin homology and RhoGEF domain containing G3 (Plekhg3), and phosphatidylinositol-4,5-bisphosphate 4-phosphatase 1 (PIP4P1). Furthermore, we found increased expression of genes playing crucial roles for lipid composition of MVs, including galactosidase alpha, glucosylceramidase beta, and *SPHK1* encoding for sphingosine kinase 1.

### Gene Ontology Enrichment Analysis

Among the altered processes in C5a-treated neutrophils, we found MV- relevant processes, such as catabolic and metabolic processes of glycosyl- and glyco-lipids, phospholipids and ceramids as well as activation and regulation of small GTPases (Supplemental Table 1). Further MV-relevant changes in pathways involve cytoskeleton-based processes (31), cell shape changes, and MAPK activity (32) (Supplemental Table 1).

### Influence of the C5aR1-Antagonist PMX53 on C5a-Mediated MV Shedding in PMNs

**Figure 4:**
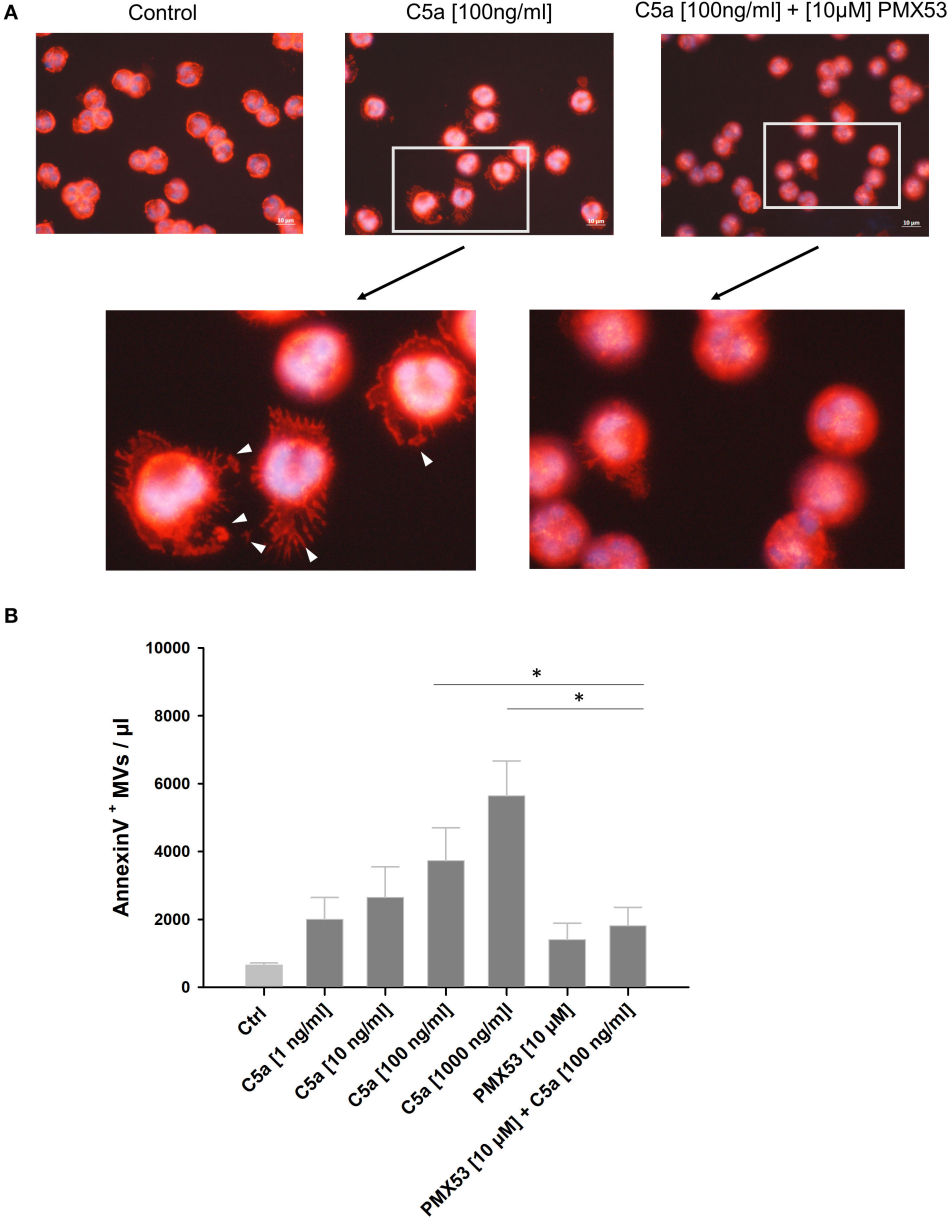
Effect of the C5aR1-antagonist PMX53 on vesicle shedding. **(A)** Upper panel shows merged image of F-actin staining (red, Phalloidin-Alexa Flour 568) and nuclei staining (DAPI) of fixed PMNs, untreated, after treatment with native human C5a (100 ng/ml), and C5a treatment in combination with PMX53 (10 μM) for 1 h, respectively at 630x magnification. Lower panel with further zoom-in on PMNs from the upper panel, white arrows indicate shed MVs. Experiment was repeated at least three times. **(B)** Quantitative analysis of shed MV at different C5a concentrations and in presence of the PMX53 together and alone in 10 μl supernatant (*n* = 3). *p < 0.05.

**Figure 5:**
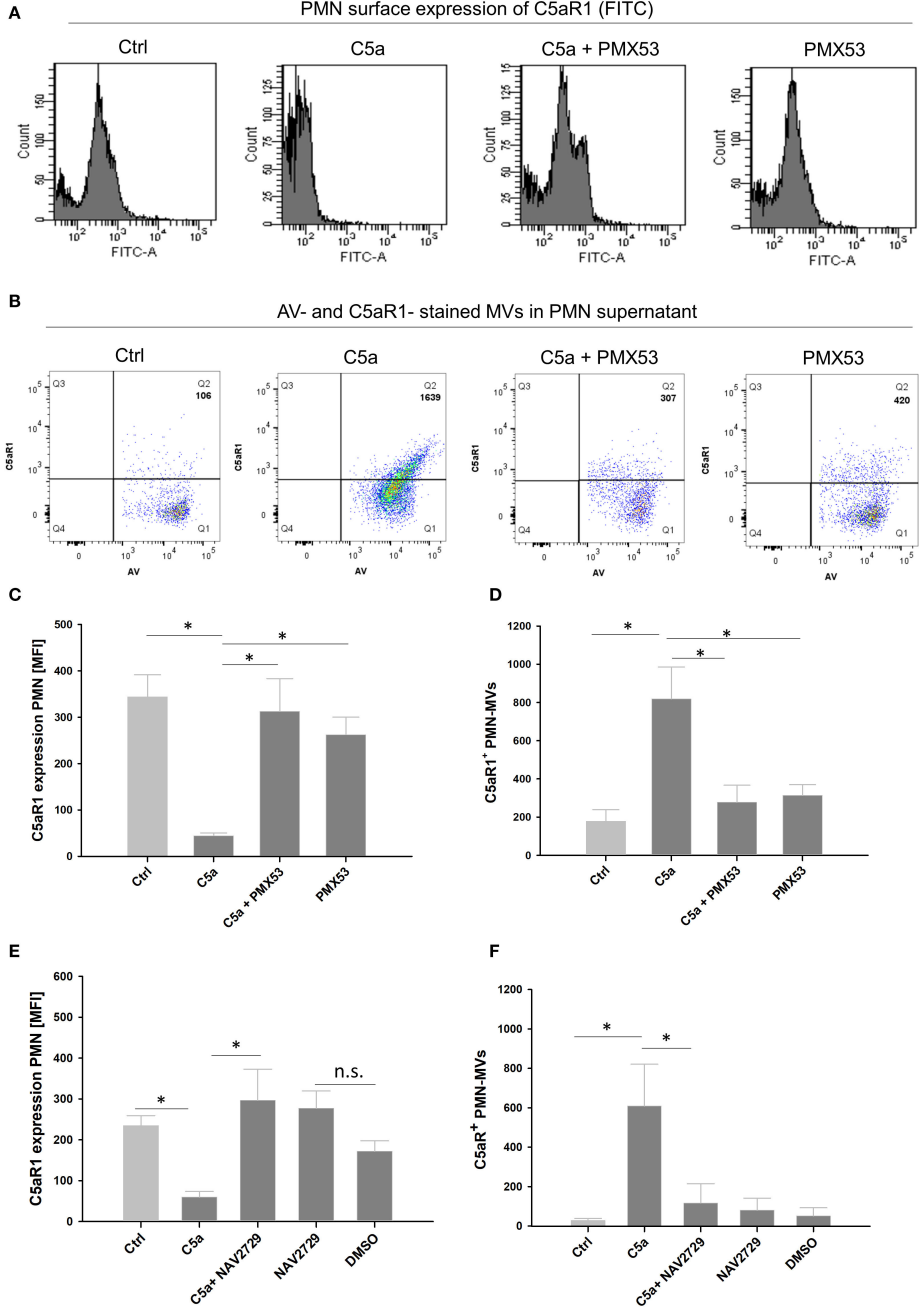
PMX53 and NAV2729 show similar effects in blocking C5aR1-positive MV shedding. Representative flow cytometry panels for C5aR1 expression (FITC) **(A)** on PMNs or **(B)** in PMN supernatants including C5aR1^+^ MV counts in Q2 after 1 h stimulation in the presence of PMX53. Quantitative analysis of flow cytometry data showing **(C)** C5aR1 expression on PMN surfaces (FITC MFI) and **(D)** C5aR1^+^ MV counts. Quantitative flow cytometry analysis including the MV-specific inhibitor NAV2729 (10 μM) **(E)** for C5aR1 expression (FITC MFI) on PMNs and **(F)** C5aR1^+^ MV counts in 10 μl PMN supernatants after 1 h, *n* = 4 per group, respectively. * *p* < 0.05.

To investigate whether the observed C5a effects were mediated by C5aR1, human neutrophils were treated with the C5aR1-specific antagonist PMX53 (33, 34). Cytoskeleton staining shows morphological changes including reorganization of actin cables into filopodia and cell spreading with vesicle-resembling structures after C5a stimulation, which were almost absent in the presence of PMX53 (Figure 4A). Quantitative analysis of the supernatants confirmed a dose-dependent increase of MV generation induced by C5a, which was blocked by PMX53 (Figure 4B). Flow cytometric analysis of C5aR1 surface expression on PMNs showed that PMX53 completely blocked the C5a-induced loss of the C5aR1 through MV shedding (Figures 5A–D). To discover a potential link between C5aR1 signaling and MV shedding, we used the Arf-6-selective inhibitor NAV2729 since Arf6 activity is described in MV formation (35). Indeed, NAV2729 was able to completely block C5a-mediated loss of C5aR1 signal on PMNs and the shedding of C5aR1^+^ MVs as shown in Figures 5E,F.

### Incubation of Human Neutrophils With Serum From Multiple Injury Patients Induces Loss of C5aR1

**Figure 6:**
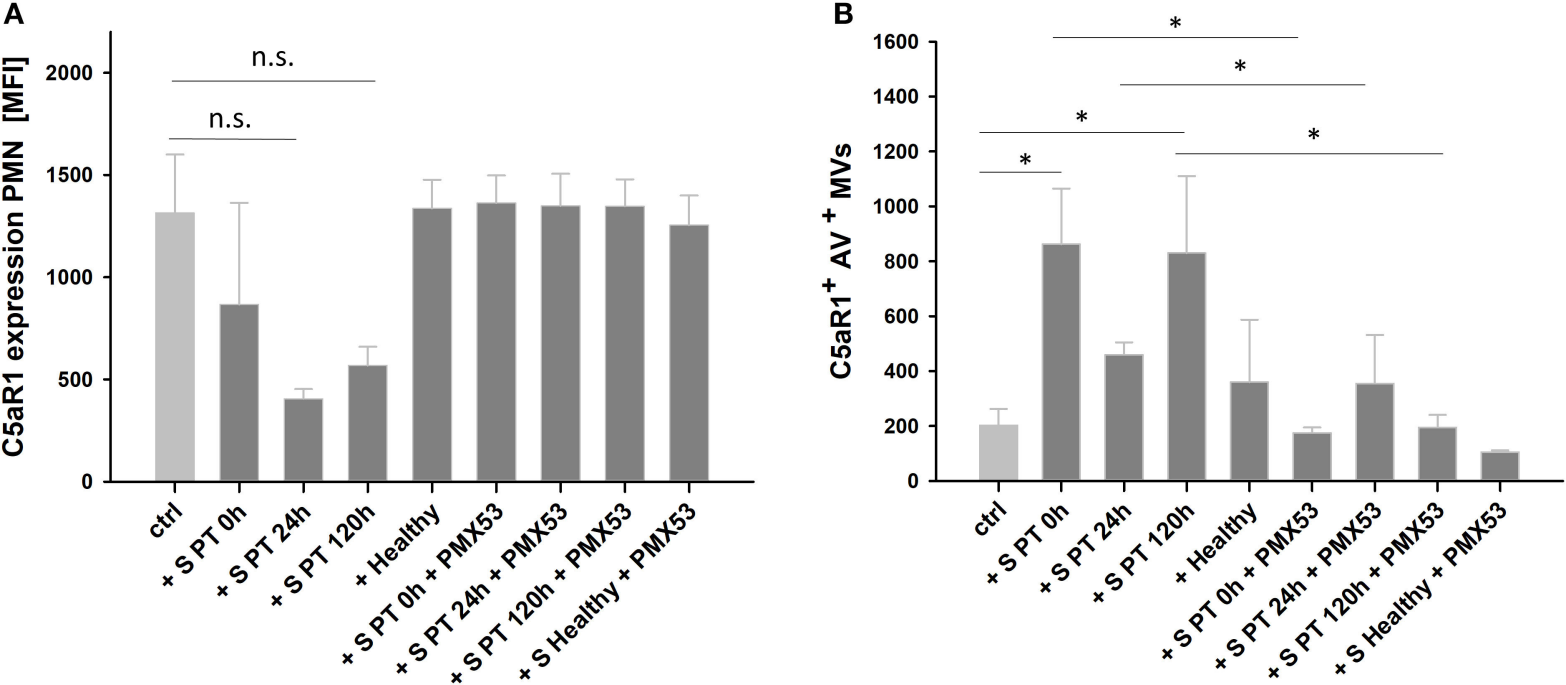
C5aR1 expression and MV shedding after incubation of PMNs with serum obtained from PT patients (S PT) and healthy donors (S Healthy). PMNs were incubated in HBSS++ buffer supplemented with 20% of pooled human serum from PT patients or from healthy donors for 1 h. **(A)** Quantification of C5aR1 expression on PMNs and **(B)** C5aR1^+^ MV counts in 10 μl of PMN supernatants after incubation. *n* = 4, *p < 0.05.

To verify the C5a-mediated effects on neutrophils, we further incubated human PMNs from healthy donors in HBSS++ buffer containing 20% MV-free serum from polytraumatized patients. We observed a reduction of the C5aR1- signal on PMNs. Moreover, we included PMX53 before adding serum to PMNs and blocked C5aR1 reduction on PMNs (Figure 6A). MV analysis in supernatants revealed that loss of PMN C5aR1 was due to shedding of C5aR1^+^ MVs, which again was blocked by PMX53 and which was nearly absent when incubated with healthy serum (Figure 6B). Since complement is a heat- labile system (36), we also incubated PMNs with heat-inactivated serum and again could block loss of the C5aR1 (Supplemental Figure 3A). Carboxypeptidases, which are present in serum can further process C5a to desarginated C5a (C5a^desArg^) (37), which can also induce cell responses via C5aR1 (38). Therefore, we additionally incubated isolated PMNs with 100 ng/ml human C5a^desArg^ and found comparable results to native human C5a (Supplemental Figures 3B,C).

### Inflammatory Features of C5a-Generated PMN-MVs

**Figure 7:**
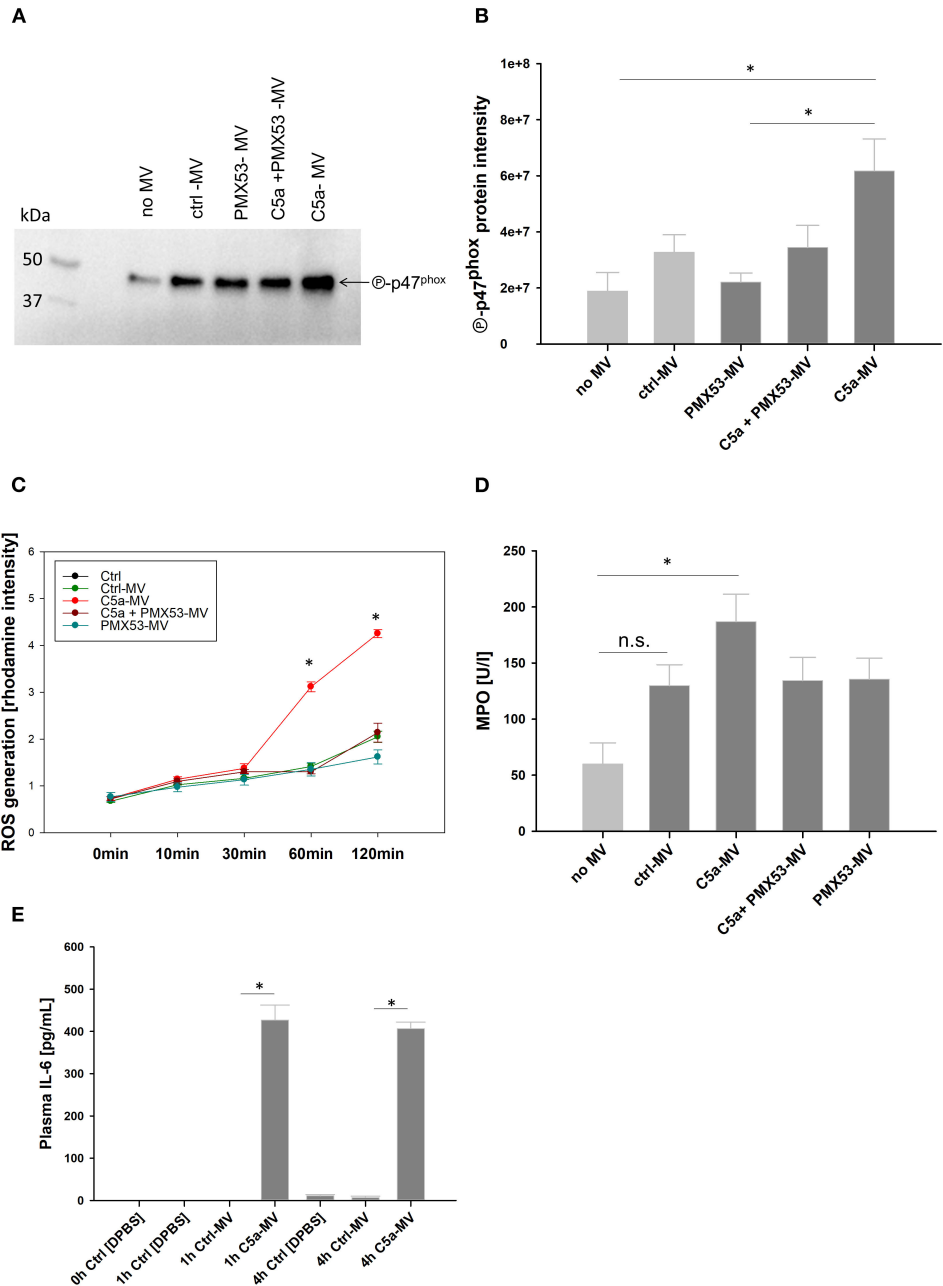
Inflammatory feature of PMN-derived MVs. **(A)** One representative immunoblot probed for phospho-p47^phox^ of whole cell lysates from PMNs. PMNs were treated with C5a, C5a and PMX53, and PMX53 only. Untreated PMNs served as control. Afterwards, PMNs from autologous donors were incubated with the generated MVs for 1h at 37°C. PMNs without MV stimulation served as control. **(B)** Quantification of phospho-p47^phox^ signal intensity of PMNs whole cell lysates from three donors. *p < 0.05 compared to PMN without MV control. **(C)** Time-dependent ROS generation in PMNs incubated with MVs, *n* = 3, *p < 0.05. **(D)** MPO amount in PMN supernatants after incubation with MVs, *n* = 3 *p < 0.05. **(E)** C5a-gerenerated MVs were incubated in whole blood from autologous donors for 1 h and for 4 h, respectively. IL-6 was determined in plasma by ELISA. *n* = 5 per group, *p < 0.05.

With regard to the functional effects of C5a-generated MVs for inflammation, we assessed central antimicrobial neutrophil functions including myeloperoxidase (MPO) release and reactive oxygen species (ROS) generation. For this purpose, resting PMNs were incubated with C5a-generated MVs from autologous donors. MVs derived from PMNs after C5a stimulation induced activation of p47^phox^, a key component of the NADPH oxidase (Figures 7A,B). As a result, C5a-generated MVs induced a significant increase in ROS production in PMNs (Figure 7C). Furthermore, MPO secretion was significantly increased in the presence of C5a-MVs (Figure 7D). To further characterize the impact of MVs on inflammation, we incubated whole blood with the C5a-generated MVs for 1 and 4 h and detected a significant increase of the secreted pro-inflammatory cytokine IL-6 at both time points compared to control MVs (Figure 7E). Western blotting revealed no residual C5a in MV preparations, ruling out a direct contribution of C5a to the observed pro-inflammatory effects (Supplemental Figure 4).

## Discussion

Trauma induces tissue injury, characterized by disruption of micro-and macrobarriers, which releases danger-associated molecular patterns (DAMPs) including histones, DNA, and HMGB1 from mechanically damaged or necrotic cells. Furthermore, loss of barrier integrity exposes the host to pathogen-associated molecular patterns including LPS. As first line of defense, the complement system promptly initiates a complex cascade of activation pathways resulting in early generation of anaphylatoxins (39). Immune complexes initiate activation of the classical pathway of complement (40, 41), while foreign surfaces and pathogen-specific proteins activate the lectin (42) and alternative pathway (43). Moreover, acidic environments due to tissue hypoperfusion have been described to activate the alternative pathway of complement (44). Besides the three established pathways of complement activation, extensive crosstalk between the complement and the coagulation system further activates complement via thrombin generation (45). Both systems are further triggered by surgical interventions and several biomaterials implanted for fracture care (46).

**Figure 8:**
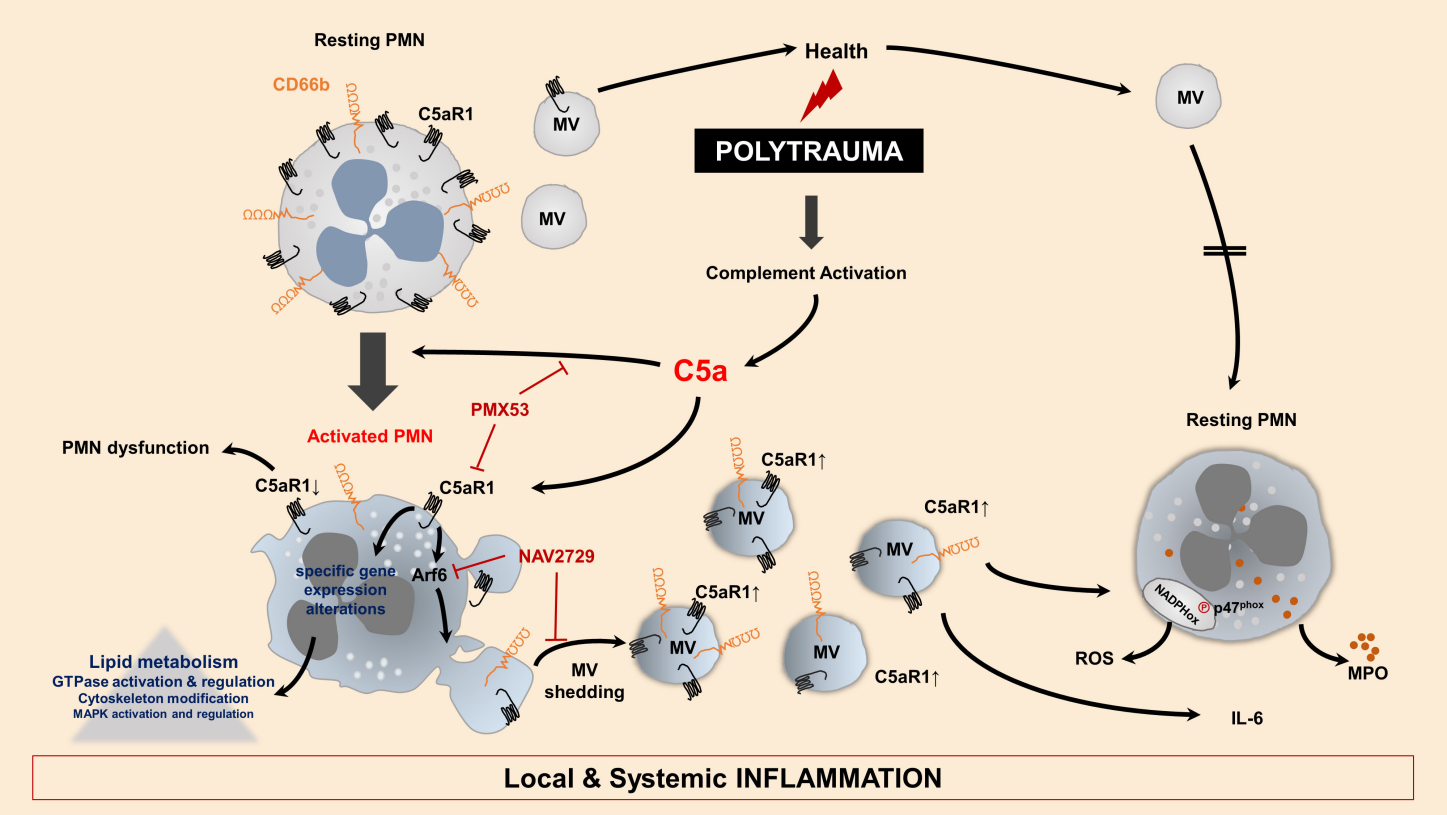
Model for C5a-mediated alterations on MV shedding for neutrophils after PT. PT causes an early activation of complement generating increased C5a concentrations. C5a induces activity of the small GTPase Arf6, which is dependent on C5aR1 signaling. Additionally, specific changes in gene expression pattern are involved in MV shedding. Resting PMNs show physiological MV shedding and composition, which have no inflammatory potential. In contrast, PMNs incubated with C5a loose C5aR1 surface expression induced by C5aR1 signaling and Arf6-mediated shedding of C5aR1-positive MVs. Furthermore, these MVs show increased CD66b and C5aR1 expression, which show pro-inflammatory features when incubated with resting PMNs. C5a induces shedding of MVs, which lead to NAPDH oxidase activation, ROS generation and MPO release in resting PMNs. Moreover, IL-6 generation was observed when these MVs were incubated in whole blood. PMX53 and the Arf6-selective compound NAV2729 can block the shedding of C5aR1-positive MVs from PMN surfaces and its inflammatory effects.

The anaphylatoxin C5a is widely described for detrimental effects in the context of inflammation and trauma. In neutrophils, C5a induced significant morphological changes including calcium-independent actin polymerization, ruffling, and cell polarization (47). In a previous study, we have shown changes in the cell shape by increased length and decreased width, decreased cell circularity, and enhanced deformability (10). Besides changes in cellular morphology, C5a is also described to enhance neutrophil adhesion to endothelial and epithelial cells (48). Especially, high C5a levels are associated with neutrophil dysfunction, resulting in impaired immune responses including chemotaxis (14) and phagocytosis (4). Moreover, high concentrations of C5a (0.5–2 μg/ml) have prevented caspase-9 activity and thus delayed apoptosis in neutrophils (49, 50). To date, C5a-induced loss of the C5aR1 on neutrophils is suspected to be responsible for post-traumatic cell paralysis and thus, among other factors, could explain the high susceptibility of trauma patients to infectious complications (14, 15). The role of C5aR2 still remains unclear and is controversially discussed in the literature. While a study showed that C5aR2 may act as negative regulator of C5a-C5aR1-mediated responses (51), another study described pro-inflammatory features of C5aR2 in C5a-primed neutrophils (52). In our study, C5aR2 expression on PMNs was unaltered after PT during the early phase after injury; in addition, C5a signaling inducing MV-dependent C5a complement receptor shedding was specifically mediated via the C5aR1, suggesting that C5aR1 is the main receptor involved in mediating C5a-induced neutrophil dysfunction. This highly specific role of C5a in PMN MV shedding is also supported by our data: increased C5aR1^+^ MV generation and loss of C5aR1 from neutrophils after stimulation with PT serum was completely abolished after C5aR1 blockade; furthermore, stimulation of PMN with trauma-relevant concentrations of other pro-inflammatory cytokines and chemokines (53) did not have any effects on MV generation or cellular C5aR1 content. Furthermore, we point to a promising therapeutic strategy since PMX53, a potent C5aR1 antagonist, completely blocked shedding of C5aR1-positive MVs (Figure 8). The pharmacological profile of PMX53 have been extensively studied in several animal models including mice (54) and rats (55). Also in humans, oral and topical administration was found to be safe and well-tolerated and successfully completed phase I clinical trials (33). Currently, PMX53 is evaluated in clinical studies addressing inflammatory disorders including patients with rheumatoid arthritis and psoriasis (56). In trauma patients, no clinical data is available so far. Considering an administration time point after PT, early application of PMX53 (at the 0 h time point), could provide a therapeutic benefit in future clinical studies.

Changes in circulating MV levels and their cellular origin have previously been described in inflammatory conditions including trauma and sepsis (57). The cellular and tissue origin of circulating MVs can provide important insights into disease pathogenesis. Severe injury causes changes of MV phenotypes including increased shedding of platelet-, erythrocyte-, endothelial-, and monocyte-derived MVs, which are able to promote coagulation (58, 59) and inflammation in trauma (57, 60). Moreover, erythrocyte-derived MV have been shown to activate pulmonary endothelial cells by increase of ELAM-1 and ICAM-1 expression as well as leukocyte-recruitment into the lung, which aggravated lung injury (61). Although elevated numbers of granulocyte-derived MVs after trauma, major burn, and sepsis have been reported (24, 62, 63), there is still little knowledge about their role in trauma and sepsis. Since neutrophil dysfunction has been observed early after injury in trauma patients, we focused on neutrophil-derived MVs and further characterized their inflammatory features by functional PMN assays. There is emerging evidence that complement activation products can influence MV shedding while simultaneously changing their features, which can boost an imbalanced inflammatory responses (64). In the present study, we demonstrated for the first time a direct mechanistic link between complement C5a and the MV shedding profile in neutrophils. These effects are likely of importance for the observed neutrophil dysfunction on the one hand and for aggravation of inflammation after multiple injury on the other hand. As in our previous study in a different trauma cohort (5), we found a post-traumatic loss of the C5aR1 on human neutrophils compared to healthy volunteers starting during the first 24 h after injury. Furthermore, we observed a clear and marked elevation of circulating granulocyte-derived MV numbers in polytraumatized patients. However, the relatively small number of patients did not allow us to perform reliable correlation analyses; for that reason, future studies need to include a larger number of patients to assess a possible connection between increased post-traumatic C5a plasma levels (1) and an increase in PMN-derived C5aR1 positive MVs. Moreover, we observed a higher variability in MV counts in PT patients, which could be explained by the high variability in the clinical cohort including injury mechanism, pattern, and severity as well as the extent of blood loss and need for transfusion, which could all influence the number of MVs (Table 1).

Furthermore, it is well-established that MV budding depends on ADP-ribosylation factor 6 (Arf6), regulating selective recruitment of proteins into MVs, and promoting MV release (35, 65). Expression of a constitutively active form of Arf6 also induces a significant increase in RhoA activity, both resulting in significantly increased MV formation (66). We were able to block both MV secretion and loss of surface C5aR1 by specific blockade of Arf6, demonstrating a so far unknown interaction between C5aR1 signaling and Arf-6-mediated downstream effects in MV shedding (Figure 8).

Besides Arf6, other members of small GTPases are also involved in vesicular trafficking and MV formation. Both cargo sorting and MV shedding are tightly regulated by several small GTPases, including members of the Arf (Arf6 and Arf1), Rab, and Rho (Rac1 and RhoA) families (35, 67). To unravel intracellular events following activation of the C5a/C5aR1 axis, we performed whole genome microarray analyses. Although we could not find changes in the expression pattern of Arf proteins, we detected significant alterations in other small GTPases including *RHOQ* and *RRAGC*, also known to be involved in MV generation. We also found significantly increased expression of Arf-like (Arl) genes including Arl11, which belong to another family of small GTPases and are involved in regulation of vesicular transport, membrane trafficking, and cytoskeletal remodeling (68). The GTP-GDP cycle in small GTPases is tightly regulated by guanine nucleotide exchange factors (GEFs) (69). We identified significant changes in GEF expression, which are involved in regulating GTPases and thus downstream MV shedding. Furthermore, we observed significant upregulation of *PIP4P1* expression after C5a stimulation, a known interaction partner of Arf6 and MV formation (70). MVs possess a specific lipid composition enriched in phospholipids including PS, phosphatidylcholine, and sphingolipids (71), which seems to critically promote uptake by recipient cells (72). In this context, we found changes in expression of *GLA, GBA*, and *SPHK1* encoding for proteins which can act to regulate the lipid composition in the plasma membrane, playing a crucial role in sphingo- and phospholipid metabolism (73). *SPHK1* is also described to be involved in sorting of vesicle cargoes of extracellular vesicles including MVs and exosomes (74, 75). A future lipidomic approach might reveal here more mechanistic insights.

Beyond the functional activation, our study reveals that C5a-induced MVs possess pro-inflammatory features and are able to activate resting neutrophils by inducing NADPH oxidase activity, ROS production, and MPO release as central mechanisms in the defense against bacterial and fungal pathogens. As a translational effort, we were able to demonstrate significant IL-6 generation in an *ex vivo* whole-blood model after addition of C5a-induced MVs. Besides monocytes and macrophages, mast cells and basophils (76), antigen-presenting cells and T cells including CD4^+^ T cells (77) and T-helper 17 cells have been described to produce IL-6 during inflammation (78, 79), while neutrophils have shown limited capacity in generating IL-6 (80, 81). Concerning our *ex-vivo* whole-blood model, it can be assumed that C5a-generated MVs induce IL-6 secretion mainly by monocytes, basophils and CD4^+^ T cells. Previous studies addressing the inflammatory features of PMN-derived MVs (PMN-MVs) did not show uniform results so far (82). Some cartilage-protective features of PMN-MVs in the context of joint inflammation have been proposed (83); furthermore, PMN-MVs were shown to limit the inflammatory response in macrophages (84). However, the type of stimulus seems to influence the inflammatory properties of PMN-MVs. In the context of acute lung injury, neutrophils have been described to release MVs which can induce activation of the endothelium (85), platelets (86), and other inflammatory cells (18). In the present study, it remains unclear which component of the C5a-generated MVs may be responsible for their considerable pro-inflammatory features. Our results clearly show that C5a-generated MVs did not contain residual C5a, indicating that these pro-inflammatory effects are not C5a-mediated and rather may originate from an altered MV composition. C5a stimulation may have affected the MV cargo and composition in comparison with those released during physiologic conditions or from unstimulated granulocytes (87, 88). Furthermore, extracellular vesicles in general have a comparably large and distinct lipid composition, harboring lipid mediators such as cholesterol, sphingomyelin, phosphatidylserine, and glycosphingolipid, and this phospholipid content is proportionally richer compared to their cellular sources (89). In a previous study, we could demonstrate that *in vitro* stimulation of monocytes with LPS led to changes in their MV phospholipid composition (90). More likely, the observed shedding of C5aR1 from the cellular surface to circulating MVs could represent a compensatory mechanism of activated neutrophils in order to reduce cellular effects of C5a as well as increase circulating C5aR1, as a decoy receptor to scavenge excessive C5a. Taken together, our findings confirm the hypothesis that C5a can induce MV shedding and significantly alter inflammatory MV features through C5aR1 signaling and subsequent Arf6 involvement. Thus, our study provides a better understanding how MVs act on the pathophysiology of multiple trauma. Inhibition or reduction of neutrophil MV shedding by targeting C5aR1 may represent a promising approach in order to protect PMN functionality and restore the MV balance during systemic inflammatory conditions such as PT and sepsis.

## Data Availability Statement

The datasets presented in this study can be found in online repositories. The names of the repository/repositories and accession number(s) can be found in the article/Supplementary Material.

## Ethics Statement

The studies involving human participants were reviewed and approved by the Independent Local Ethics Committee of the University of Ulm, Helmholtzstrasse 20 (Oberer Eselsberg), 89081 Ulm, Germany. The patients/participants provided their written informed consent to participate in this study.

## Author Contributions

EK, JD, SK, and RH performed the research experiments. EK and RH planned the experimental procedures, analyzed the data, and wrote the paper. MH-L contributed to the interpretation of data. KH performed microarray analysis, analyzed, and interpreted the data. MK, SK, and FG provided PT patient samples and clinical data for analysis. CS and JK critically reviewed the manuscript. All authors read and approved the manuscript.

## Conflict of Interest

The authors declare that the research was conducted in the absence of any commercial or financial relationships that could be construed as a potential conflict of interest.

## Abbreviations

Arf6: ADP-ribosylation factor 6
AV: annexin V
C5a^desArg^: desarginated C5a
C5aR1: complement anaphylatoxin 5a receptor 1
C5aR2: complement anaphylatoxin 5a receptor 2
EDTA: ethylenediamine tetraacetic acid
GDP: guanosine-diphosphate
GEF: nucleotide exchange factors
GTP: guanosine-triphosphate
MFI: mean fluorescence intensity
MPO: myeloperoxidase
MV: microvesicles
PMN: polymorphonuclear neutrophils
PS: phosphatidylserine
PT: polytrauma
ROS: reactive oxygen species.
