# Supplementary material for: Complement C5a Induces Pro-inflammatory Microvesicle Shedding in Severely Injured Patients
Source: Front Immunol. 2020 Sep 2;11:1789. doi: 10.3389/fimmu.2020.01789 (PMC7492592; doi:10.3389/fimmu.2020.01789)
Supplement: Supplementary file 1 [file Table_1.DOCX]

**Supplemental Table 1: Regulated biological processes upon C5a exposure of neutrophils.**

|  | **Biological pathways** | **Genes** | | **Change** |  | |
| --- | --- | --- | --- | --- | --- | --- |
| **GO ID** |  | **Up** | **Down** | **[%]** | **p-value** | |
| 46477 | glycosylceramide catabolic process | 2 | 0 | 28.6 | <1×10^-3^ |  |
| 6678 | glucosylceramide metabolic process | 2 | 0 | 28.6 | <1×10^-3^ |  |
| 46479 | glycosphingolipid catabolic process | 3 | 0 | 27.3 | <1×10^-3^ |  |
| 19377 | glycolipid catabolic process | 3 | 0 | 25 | <1×10^-4^ |  |
| 6681 | galactosylceramide metabolic process | 1 | 0 | 20 | 3×10^-2^ |  |
| 6670 | sphingosine metabolic process | 2 | 0 | 20 | 2×10^-3^ |  |
| 6677 | glycosylceramide metabolic process | 3 | 0 | 20 | 2×10^-4^ |  |
| 30149 | sphingolipid catabolic process | 4 | 0 | 19 | <1×10^-6^ |  |
| 46466 | membrane lipid catabolic process | 4 | 0 | 18.2 | <1×10^-6^ |  |
| 46512 | sphingosine biosynthetic process | 1 | 0 | 16.7 | 4×10^-2^ |  |
| 32851 | positive regulation of Rab GTPase activity | 1 | 0 | 16.7 | 4×10^-2^ |  |
| 19374 | galactolipid metabolic process | 1 | 0 | 16.7 | 4×10^-2^ |  |
| 46514 | ceramide catabolic process | 3 | 0 | 16.7 | 3×10^-4^ |  |
| 46519 | sphingoid metabolic process | 2 | 0 | 15.4 | 3×10^-4^ |  |
| 31941 | filamentous actin | 2 | 0 | 10 | 9×10^-3^ |  |
| 30239 | myofibril assembly | 3 | 0 | 8.3 | 2×10^-3^ |  |
| 19003 | GDP binding | 1 | 1 | 7.4 | 1×10^-2^ |  |
| 5884 | actin filament | 3 | 1 | 6.9 | 8×10^-4^ |  |
| 6687 | glycosphingolipid metabolic process | 4 | 0 | 6.7 | 1×10^-3^ |  |
| 31032 | actomyosin structure organization | 3 | 0 | 6.5 | 4×10^-3^ |  |
| 6672 | ceramide metabolic process | 4 | 0 | 6.3 | 1×10^-3^ |  |
| 6665 | sphingolipid metabolic process | 5 | 0 | 4.4 | 1×10^-3^ |  |
| 6664 | glycolipid metabolic process | 4 | 0 | 4.0 | 6×10^-3^ |  |
| 8360 | regulation of cell shape | 2 | 2 | 4.0 | 6×10^-3^ |  |
| 6643 | membrane lipid metabolic process | 5 | 0 | 3.2 | 5.5×10^-3^ |  |
| 5088 | Ras guanyl-nucleotide exchange factor activity | 2 | 1 | 2.8 | 4×10^-2^ |  |
| 44242 | cellular lipid catabolic process | 4 | 0 | 2.8 | 2×10^-2^ |  |
| 30016 | myofibril | 3 | 1 | 2.7 | 2×10^-2^ |  |
| 43405 | regulation of MAP kinase activity | 6 | 0 | 2.5 | 8×10^-3^ |  |
| 43406 | positive regulation of MAP kinase activity | 4 | 0 | 2.3 | 3×10^-2^ |  |
| 5085 | guanyl-nucleotide exchange factor activity | 3 | 1 | 2.2 | 4×10^-2^ |  |
| 5083 | small GTPase regulator activity | 4 | 1 | 1.8 | 4×10^-2^ |  |
|  |  |  |  |  |  |  |

Abbreviations: GDP, guanosine diphosphate; GO ID, gene ontology identifier; GTPase, guanosine triphosphate binding protein; RabGTPase, Ras-related in brain GTPase
