# Supplementary figures and images for: Complement C5a Induces Pro-inflammatory Microvesicle Shedding in Severely Injured Patients
Source: Front Immunol. 2020 Sep 2;11:1789. doi: 10.3389/fimmu.2020.01789 (PMC7492592; doi:10.3389/fimmu.2020.01789)

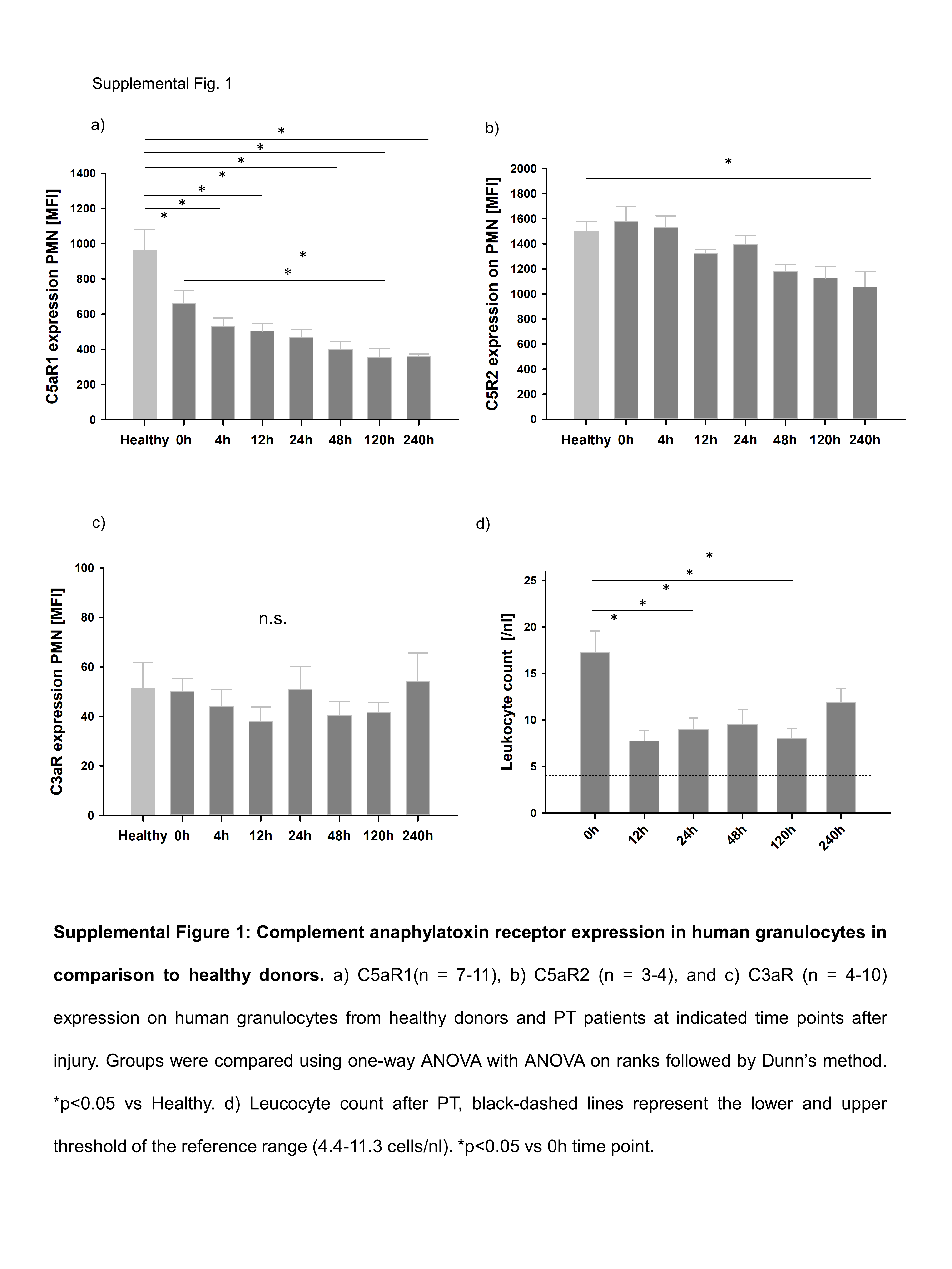

Supplement: Supplementary file 2 [file Image_1.TIF]

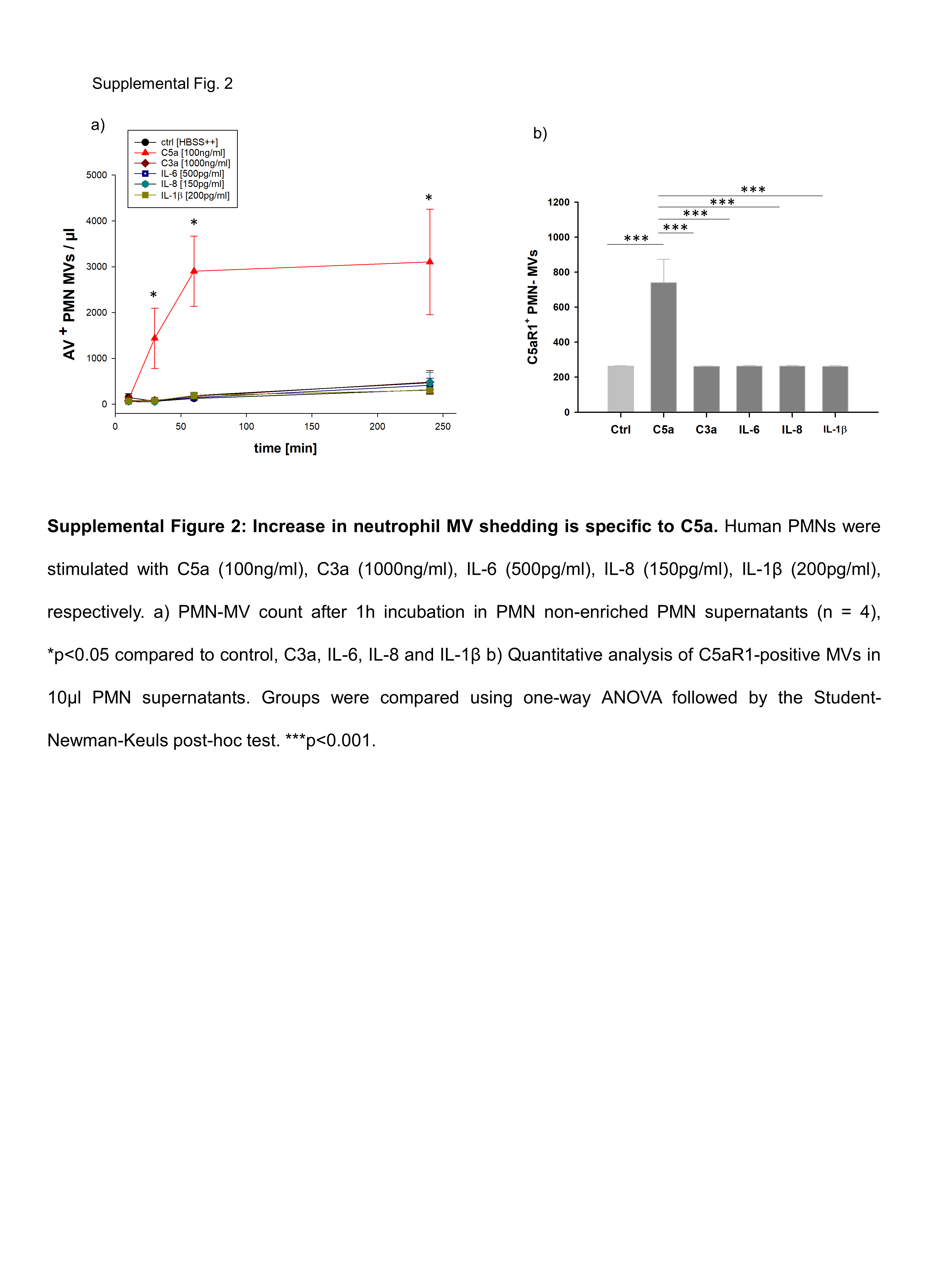

Supplement: Supplementary file 3 [file Image_2.TIF]

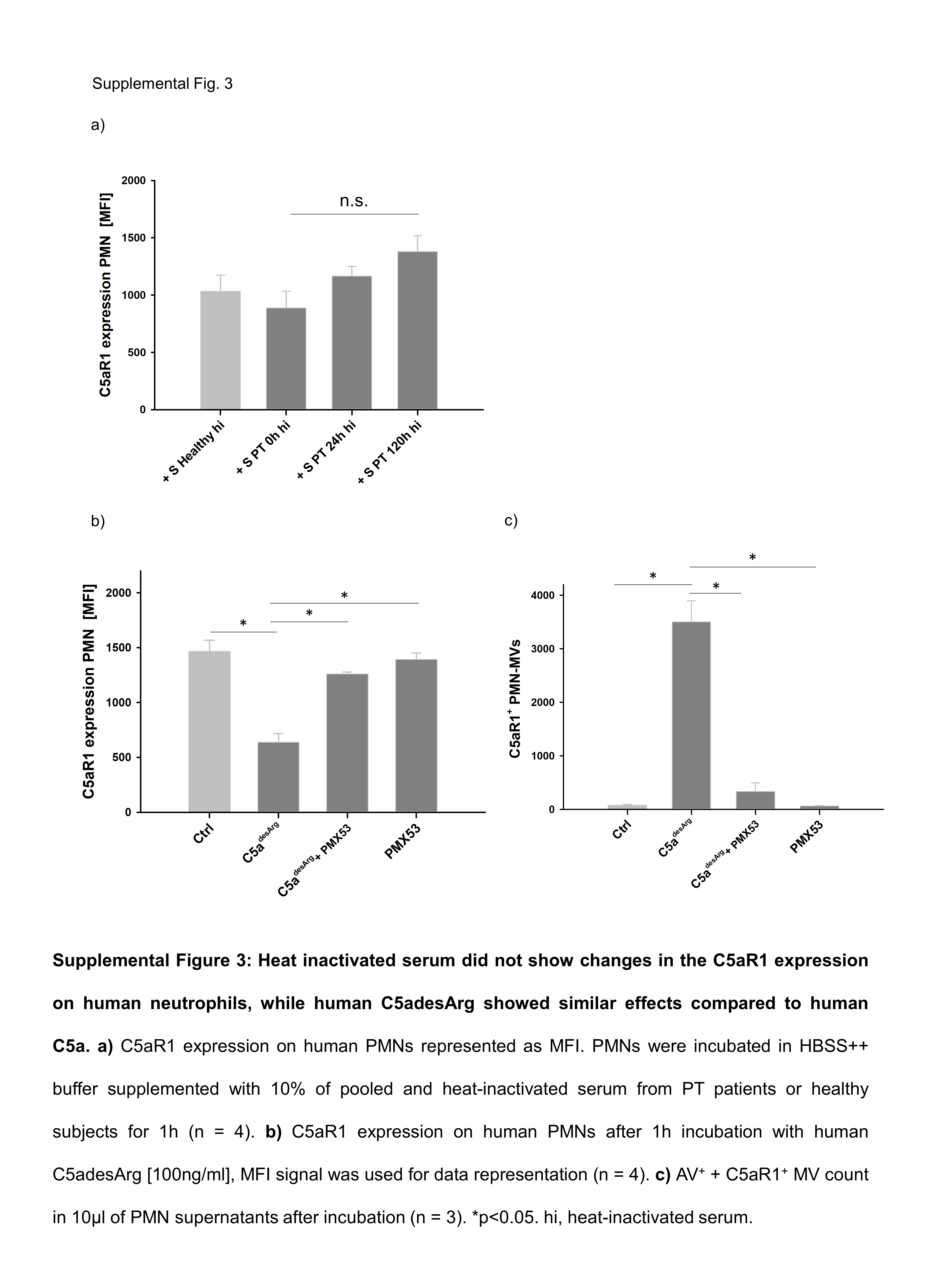

Supplement: Supplementary file 4 [file Image_3.TIF]

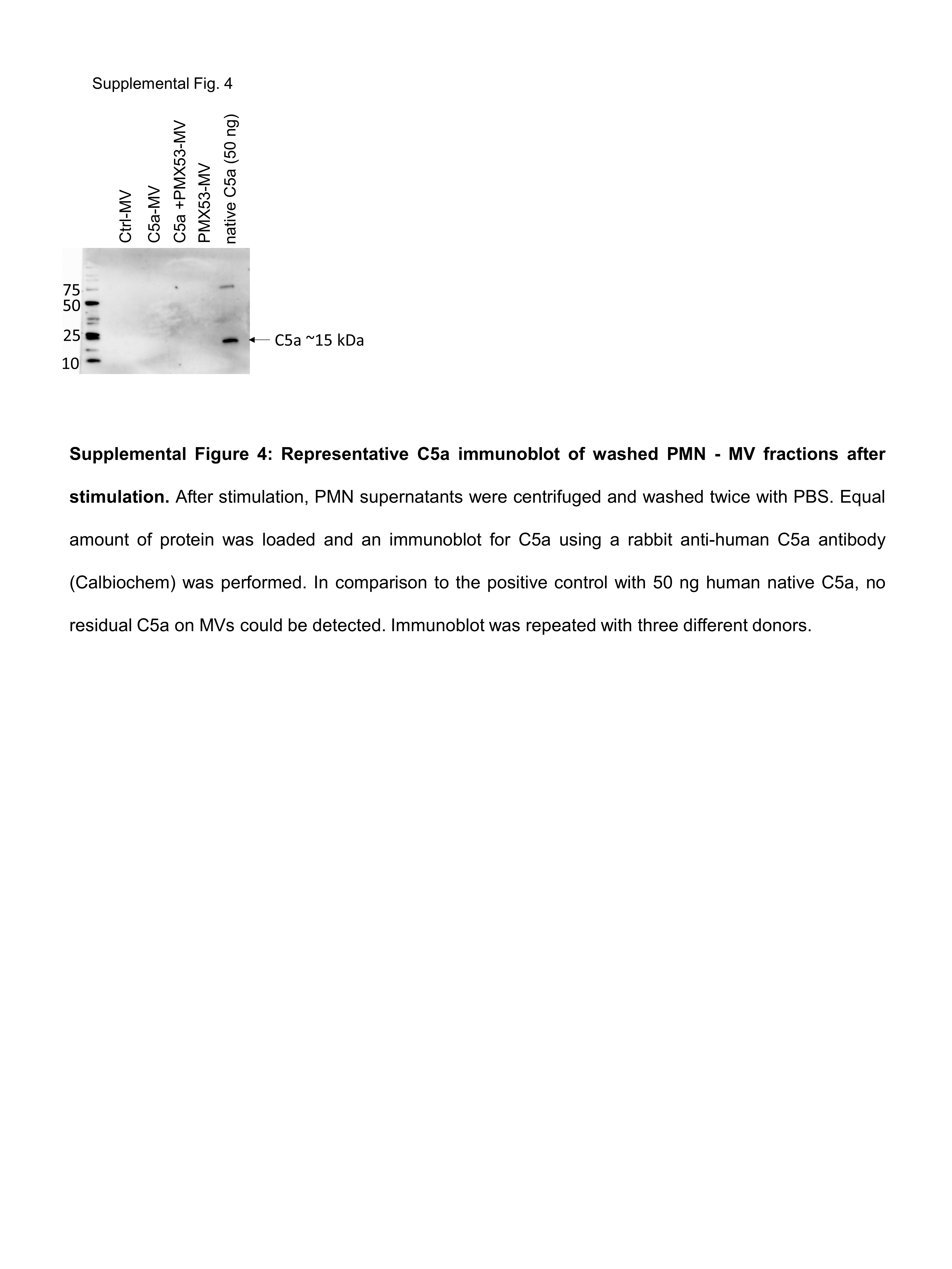

Supplement: Supplementary file 5 [file Image_4.TIF]
